# Influencing Factors of Thermogenic Adipose Tissue Activity

**DOI:** 10.3389/fphys.2016.00029

**Published:** 2016-02-05

**Authors:** Guoqing Zhang, Qinghua Sun, Cuiqing Liu

**Affiliations:** ^1^Department of Occupational and Environmental Health, Dalian Medical UniversityDalian, China; ^2^Basic Medical College, Zhejiang Chinese Medical UniversityHangzhou, China; ^3^Division of Environmental Health Sciences, College of Public Health, Ohio State UniversityColumbus, OH, USA

**Keywords:** thermogenesis, brown adipose tissue, brite/beige adipocytes, obesity, air pollution

## Abstract

Obesity is an escalating public health challenge and contributes tremendously to the disease burden globally. New therapeutic strategies are required to alleviate the health impact of obesity-related metabolic dysfunction. Brown adipose tissue (BAT) is specialized for dissipating chemical energy for thermogenesis as a defense against cold environment. Intriguingly, the brown-fat like adipocytes that dispersed throughout white adipose tissue (WAT) in rodents and humans, called “brite” or “beige” adipocytes, share similar thermogenic characteristics to brown adipocytes. Recently, researchers have focused on cognition of these thermogenic adipose tissues. Some factors have been identified to regulate the development and function of thermogenic adipose tissues. Cold exposure, pharmacological conditions, and lifestyle can enhance non-shivering thermogenesis and metabolism via some mechanisms. However, environmental pollutants, such as ambient fine particulates and ozone, may impair the function of these thermogenic adipose tissues and thereby induce metabolic dysfunction. In this review, the origin, function and influencing factors of thermogenic adipose tissues were summarized and it will provide insights into identifying new therapeutic strategies for the treatment of obesity and obesity-related diseases.

## Introduction

According to a systematic analysis, there were more than 2.1 billion obese or overweight people worldwide in 2013 (Ng et al., 2014). Substantial evidence implicates that obesity can lead to inflammation, cellular dysfunctions, and ultimately obesity-related complex metabolism disorders or cardiovascular diseases, even certain types of cancer (Flegal et al., 2013; Wali et al., 2014). Thus, obesity is an escalating public health challenge and a major cause of disease burden globally.

It is well known that there are mainly two kinds of adipose tissues in mammals: white adipose tissue (WAT) and brown adipose tissue (BAT). WAT is the major site for energy storage and secretion of adipokines which are involved in metabolic processes, such as adiponectin, leptin, TNFα, etc. (Alemany, 2013). In contrast, BAT is specialized for dissipating chemical energy for thermogenesis that is mediated by the uncoupling protein 1 (UCP1) present in the inner mitochondrial membrane (Ricquier, 2005). This process is termed as non-shivering thermogenesis. Studies implicate that BAT activation improves insulin sensitivity and is positively associated with resistance of obesity and metabolic diseases (Gasparetti et al., 2003; Labbe et al., 2015). Additionally, recent studies have demonstrated that some “brown-like” cells dispersed throughout WAT and expressed high levels of *UCP1* (Wu et al., 2012). These cells are called “beige” adipocytes (Wu et al., 2012) or “brite” (brown in white; Walden et al., 2012). Due to the heat production of BAT and browned WAT, these adipose tissues are defined as thermogenic adipose tissues. In fact, thermogenic adipose tissue is a dynamic organ, with metabolic phenotype varying dramatically in response to environment factors, pharmacological conditions, and lifestyle. In this review, the recent cognition of the thermogenic adipose tissues and the relevant evidence on their influencing factors were summarized.

## Characteristics of thermogenic adipose tissues

### Characteristics of BAT

Morphologically, BAT exhibits a dense vascularity and is mainly composed of brown adipocytes, which is characterized by multiple small cytoplasmic lipid droplets and a large number of mitochondria that are spherical, large, and packed with laminar cristae (Table 1; Cinti, 2009). Functionally, BAT plays a pivotal role in non-shivering thermogenesis which is highly regulated by the sympathetic nervous system. UCP1, located in the inner mitochondrial membrane, separates oxidative phosphorylation from ATP synthesis with energy dissipated as heat, and thereby contributing to thermogenesis (Table 1; Ricquier, 2005). Structurally, the 5'-flanking region of the *UCP1* gene includes a proximal regulatory region and an upstream enhancer. The proximal regulatory promoter includes CCAAT-enhancer binding protein (C/EBP)-regulated sites and a cAMP-regulatory element. The upstream enhancer has a complex organization of nuclear receptor binding sites called peroxisome proliferator response element that can bind either co-activating the peroxisome proliferator-activated receptor γ (PPARγ) or co-activating the peroxisome proliferator-activated receptor α (PPARα), both of which have been identified in regulating lipid metabolism and thermogenesis (Barbera et al., 2001; Santos et al., 2015).

**Table 1:** **Major differences between brown adipocytes and beige/brite adipocytes**.

**Types**	**Morphological features**	**Developmental ancestry origin**	**Distribution in rodents**	**Distribution in humans**
Brown adipocytes	Multiple small cytoplasmic lipid droplets A large number of spherical and large mitochondria which are packed with laminar cristae High expression of UCP1	*Myf5*-positive cells	Interscapular regions	Supraclavicular Anterior neck Paraspinal regions Mediastinal Para-aortic Suprarenal regions
Beige/brite adipocytes	Multilocular adipocytes Highly mitochondrial content Low expression of UCP1	white adipocytes Sca-1^+^ progenitor cells Smooth muscle cell progenitors PDGFRα^+^ cells	Inguinal subcutaneous WAT > epidydimal WAT Epicardial adipose tissue	Subcutaneous WAT > mesenteric or omental WAT

The existence of BAT was well documented in rodents and human. In rodents, the most prominent and greatest amount of BAT was localized in the interscapular depot (Nedergaard et al., 2007), whereas human BAT depots were being found in the supraclavicular, anterior neck, paraspinal regions, mediastinal, para-aortic, and suprarenal regions (Table 1; Nedergaard et al., 2007; Pfeifer and Hoffmann, 2014). Human shows relatively abundant presence and high activity in BAT at birth and it plays a crucial role in defending newborns' body temperature without shivering. However, BAT rapidly regresses during postnatally periods, and significantly disappears with ages (Nedergaard et al., 2007), with its existence well documented in infants and young children but not adults (Cannon and Nedergaard, 2004). However, by detecting uptake of radioactively labeled glucose [2-deoxy-2- (^18^F)fluoro- D-glucose(FDG)] with integrated positron-emission tomography and computed tomography, recent studies indicated that adult humans also showed a significant amount of metabolically active BAT in response to cold stimuli (van Marken Lichtenbelt et al., 2009). These results indicate that the amount or activity of BAT may be dependent on some factors in adult human.

Previously, white and brown adipocytes were assumed to share a common developmental ancestry origin. However, Atit et al. showed that interscapular brown adipose tissue (iBAT) had the same developmental origin with skeletal muscle, but not WAT (Atit et al., 2006). Both brown adipocytes and muscle cells were emanated from precursors that express *myf5* (*myf5*-positive cells), a gene thought to be expressed only in the myogenic lineage (Table 1; Seale et al., 2008). Seale et al. demonstrated that the transcriptional regulator, PRD1-BF1-RIZ1 homologous domain containing 16 (*PRDM16*), induced the activation of brown adipocytes originating from *myf5*-positive cells (Seale et al., 2008) and played a critical role in the switch between myoblastic precursors and brown adipocytes by forming a transcriptional complex with the active form of C/EBP-β (Kajimura et al., 2009).

### Characteristics of beige adipocytes

It has been known for many years that some brown fat-like adipocytes, called “beige” or “brite” cells, were found dispersed throughout WAT in rodents and humans (Schulz et al., 2011; Seale et al., 2011). The development of beige cells in WAT was dramatically enhanced in response to cold or β_3_-adrenergic receptor(β_3_AR) stimulation (Cousin et al., 1992; Granneman et al., 2005). Beige cells are multilocular adipocytes, which have high mitochondrial content (Frontini and Cinti, 2010) and low expression of the thermogenic genes, including UCP1 (Cousin et al., 1992), compared with classic brown adipocytes in the basal (unstimulated) state (Table 1). However, the beige cells expressed high levels of UCP1 when they were exposed to chronic cold or stimulated by β-adrenergic agonist (Cousin et al., 1992).

Of note, beige adipocytes, which is distinct from classical brown adipocytes, were not derived from the *Myf5*-positive cells (Petrovic et al., 2010), with the origin or browning phenotype varying with different locations of WAT. For instance, inguinal subcutaneous WAT (iWAT) had a greater propensity to a brown-like phenotype (Seale et al., 2011), whereas the epidydimal WAT (eWAT) was less prone to browning (Seale et al., 2011). The majority of beige cells in subcutaneous WAT (sWAT) arose from the direct transformation of differentiated white adipocytes (Barbatelli et al., 2010) or Sca-1^+^ progenitor cells (ScaPCs, Sca-1^+^/CD45^−^/Mac1^−^; Schulz et al., 2011) or smooth muscle cell (Myh11+) progenitors (Long et al., 2014). Almost all beige cells in abdominal WAT are derived from the PDGFRα^+^ cells which express CD34, stem cell antigen 1(Sca-1), and platelet-derived growth factor receptor alpha (PDGFRα; Lee et al., 2012). Moreover, epicardial adipose tissue (EAT) is an unusual visceral fat depot with anatomical and functional contiguity to the myocardium and coronary arteries. Compared to WAT around epididymis or kidney, EAT showed much smaller adipocytes and higher expression of BAT specific genes such as *UCP1, PGC1*α (proliferator-activated receptor-γ coactivator 1α), and *Cidea*, suggesting a browning phenomenon of EAT (Sun et al., 2013). Consistent with these observations on mice, human preadipocytes isolated from sWAT were more propensity to browning than cells isolated from mesenteric or omental WAT (Table 1; Schulz et al., 2011). Interestingly, another study showed that the components of the previously described BAT depots in adult humans might not be the classical brown adipocytes, but the beige cells (Wu et al., 2012). Therefore, the identification of brown fat in adult humans, brown adipocytes, or beige adipocytes, remains controversial.

## Influencing factors of the function of thermogenic adipose tissue

Due to the energy expenditure function of thermogenic adipose tissue, activation of these adipose tissues may be a potential strategy against obesity or metabolic diseases by regulating energy balance. Elaborating the factors which influence the function of thermogenic adipose tissues is beneficial to exploring some therapeutical targets to remedy obesity-related diseases. Activation of BAT and the browning of WAT have been associated previously with temperature, medication, lifestyle, and environmental pollutants.

### Activation of thermogenic adipose tissue by cold stimulation

After birth, newborns are directly or successively exposed to cold and require postnatal thermogenesis to complement heat loss both in animals and in humans. BAT activation plays an especially crucial role in defense of body temperature in cold conditions. In this regard, the metabolic activation and energy substrate utilization *in vivo* of BAT is increased in a cold environment (19 or 10°C; Saito et al., 2009; Labbe et al., 2015). Consistent with it, Masayuki Saito et al. revealed that FDG uptake in BAT was negligible in warm conditions but markedly increased after cold exposure (19°C), and the incidence of FDG uptake into BAT showed seasonal variations, markedly increased in winter compared with summer (Saito et al., 2009). Keeping in line with it, it has been shown that exposure to cold (10–18°C) increased blood perfusion, enhanced oxidative metabolism, elevated glucose, and non-esterified fatty acid uptake in BAT (Orava et al., 2011; Ouellet et al., 2012; Labbe et al., 2015). These results suggest that the function of BAT is activated by cold exposure.

**Figure 1:**
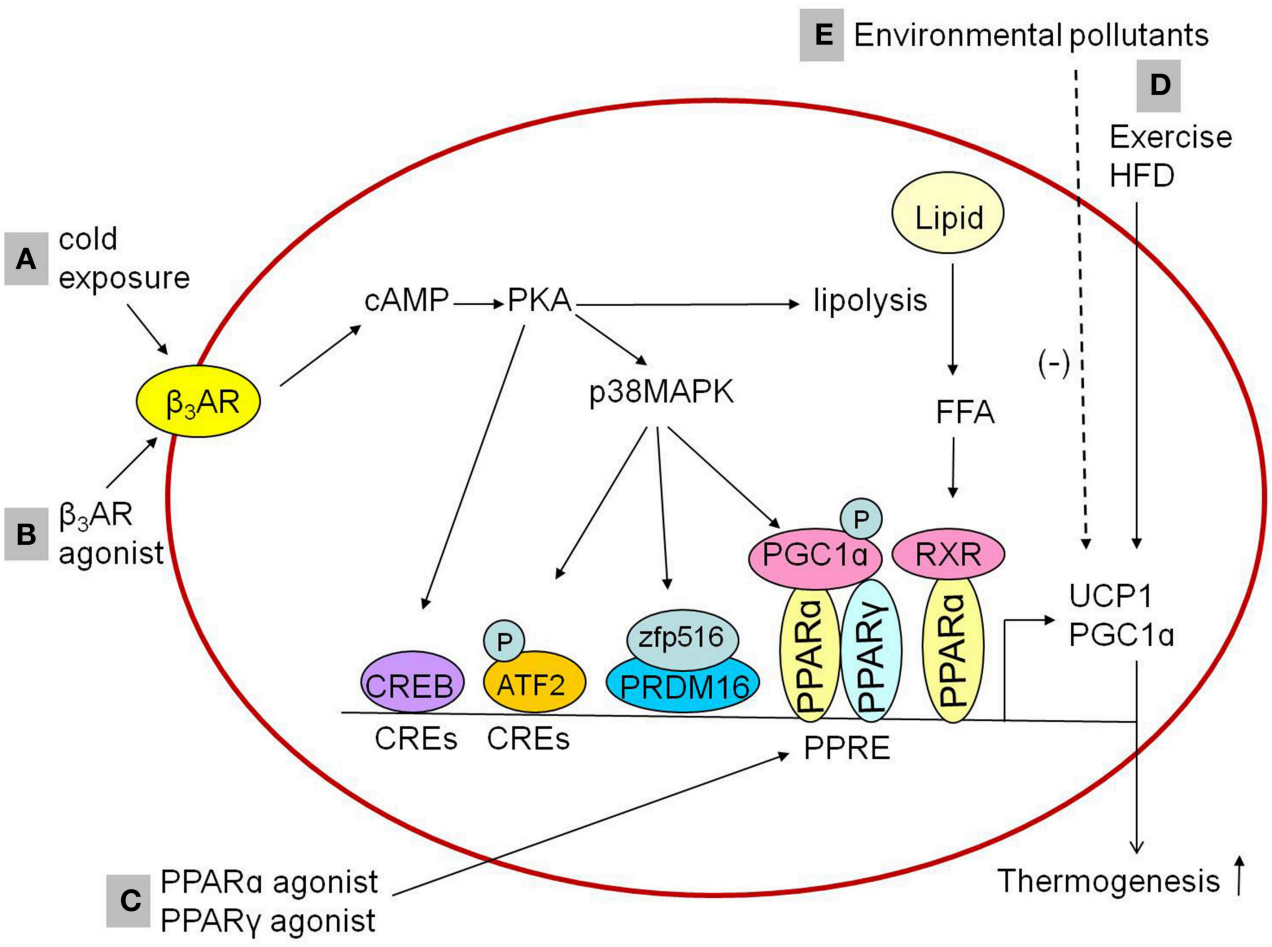
**Influencing factorsinducing thermogenesis in the BAT. (A,B)** Cold exposure and β_3_AR agonist promotes the expression of thermogenic genes in BAT through cAMP-dependent PKA actication via β_3_AR. The activated PKA might activate RXR/PPARα through the liberation of FFA from triglyceride stores and lipolysis to promote thermogenic genes expression. In addition, PKA activation could promote thermogenic genes expression both p38 MAPK-independently and p38 MAPK-dependently, in a p38 MAPK-independent way, PKA promotes the expression of thermogenic genes through CREB which bind to CREs. In a p38 MAPK-dependent way, PKA phosphorylates ATF2 and PGC1α or activates zfp516/PRDM16 interaction to induce the expression of thermogenic genes. **(C)** PPARγ agonists or PPARα agonists upregulate the expression of UCP1 and PGC1α in brown adipocytes, which is mediated by the PPAR. **(D)** Exercise and HFD feeding upregulate the expression of UCP1 in brown adipocytes. **(E)** Environmental pollutants downregulate the expression of UCP1 and PGC1α in brown adipocytes. β_3_AR, β3-adrenergic receptor; PKA, protein kinase A; RXR, retinoid X receptor; p38MAPK, p38 mitogen-activated protein kinase; FFA, free fatty acids; CREB, cAMP response element binding protein; CREs, cAMP-regulatory elements; ATF2, activating transcription factor 2; Zfp516, zinc-finger protein516; PRDM16, PRD1-BF1-RIZ1 homologous domain containing 16; PPARα, peroxisome proliferator activated receptor α; PPARγ, peroxisome proliferator activated receptor γ; PGC1α, peroxisome proliferator activated receptor-γ coactivator-1α; PPRE, peroxisome proliferator response element; UCP1, uncoupling protein 1; HFD, high-fat diet.

The mechanism by which cold stimulates BAT activation are widely explored and summarized in Figure 1A. When exposed to cold, cold sensations signal activating the sympathetic nervous system to release norepinephrine, which binds with β_3_AR, followed by catalyzing production of cyclic AMP (cAMP) and activation of cAMP-dependent protein kinase (PKA). The cold-stimulated β_3_AR-cAMP-PKA pathway induces the expression of UCP1 in BAT, followed by liberation of free fatty acids from triglyceride stores and lipolysis which might activate PPARα in complex with retinoid X receptor and upregulate expression of thermogenic genes (Collins, 2011; Bartelt and Heeren, 2012). The transcription of UCP1 promoted by PKA could be in both p38 mitogen-activated protein kinase (MAPK)-dependent and p38 MAPK-independent pathways. There are at least three factors which are activated by PKA-phosphorylated p38 MAPK. Firstly, nuclear factor activating transcription factor 2 (ATF2) could be phosphorylated by p38 MAPK and enhances transcription of the *PGC1*α and *UCP1* genes through the cAMP-regulatory elements (Cao et al., 2004). Secondly, Zfp516, a zinc-finger protein, has been shown to be induced by cold stimulation via the cAMP response element binding protein (CREB)/ATF2 pathway (Dempersmier et al., 2015). Jon Dempersmier et al. observed that p38 MAPK phosphorylation also induces interaction of Zfp516 with PRDM16 to activate the transcription of thermogenic genes such as *UCP1* (Dempersmier et al., 2015). Thirdly, the activated p38 MAPK could directly phosphorylates PGC1α protein, then promotes transcription of the *UCP1* through PPARγ bound to the *UCP1* peroxisome proliferator response element (Cao et al., 2004). The p38 MAPK-independent pathway is that PKA directly promotes transcription of the UCP1 by the transcription factor CREB, which binds to the CRE in the proximal promoter region of the *UCP1* gene (Yubero et al., 1998).

Mounting evidence suggest that exposure to cold *in vivo* induces WAT browning. The expression of *UCP1* in WAT was increased significantly in response to intermittent exposure to cold (5–6°C; Bai et al., 2015). Quantitative electron microscopy disclosed that cold exposure (6°C for 10 days) induced appearance of several “brown like” cells in visceral adipose tissue with an intermediate morphology between white and brown adipocytes, suggesting an apparent transformation of white into brown adipocytes (trans-differentiation). This process predominantly reflects β_3_AR mediated trans-differentiation which was blunted in β_3_-KOmice (Barbatelli et al., 2010). Zfp516, induced by cold exposure in BAT, caused an increase in *UCP1* expression and browning trans-differentiation in sWAT too (Dempersmier et al., 2015). Regard with it, researchers identified another regulatory mechanism that mediated this cold response. Type 2 (alternative) macrophages (M2 macrophages) were recruited to sWAT to induce catecholamine production via IL-4/13 signaling, inducing WAT browning in cold environment (4–5°C; Nguyen et al., 2011; Qiu et al., 2014). However, the detailed signaling pathways remain further investigation.

BAT has been shown to play a pivatol role in glucose homeostasis (Gasparetti et al., 2003), which is regulated by the insulin signaling pathway. Cold exposure (4°C for 4 h or 8 days) significantly up-regulated the expression and/or phosphorylation of insulin receptor, insulin substrates, and p-Akt in the BAT of rats (Gasparetti et al., 2003; Wang and Wahl, 2014). These findings suggest that cold stimulation has the potential to improve glucose clearance and insulin sensitivity by insulin signaling pathways.

### Regulation of the thermogenic process by pharmacological factors

Since sympathetic activation has been established to promote the expression of thermogenic genes in BAT, the adrenergic receptor agonist may regulate the thermogenic process through sympathetic activation-like mechanism. Consistent with the inference, it was observed that treatment with β_3_-adrenergic receptor agonist activated BAT (Cypess et al., 2015; Park et al., 2015; Figure 1) and induced browning of iWAT (Park et al., 2015). The β_3_-adrenergic receptor agonistCL316,243 (5-[(2R)-2-[[(2R)-2-(3-chlorophenyl)-2-hydroxyethyl]amino]propyl]-1,3-benzodioxole-2,2-dicarboxylic acid disodium salt) up-regulated expression of thermogenic genes such as *UCP1* in BAT or both *UCP1* and *PRDM16* in iWAT (Park et al., 2015). Thus, the β_3_-adrenergic receptor agonist such as CL316,243 may plays an important role in BAT activation and WAT browning with cAMP-dependent PKA activation as the potential mechanism (Park et al., 2015).

In addition to sympathetic stimulation, non-sympathetic stimulation-mediated regulation for the activation of BAT has also been recognized. Recently, researchers identified peroxisome proliferator-activated receptors (PPARs) as a pivotal role in the regulation of transcriptional thermogenic genes. The upstream enhancer of 5′-flanking region in the *UCP1* gene has a complex organization of nuclear receptor binding sites called “PPAR,” which can bind either PPARγ or PPARα (Barbera et al., 2001). PPARγ is required for the formation and differentiation of both brown and white adipocytes (Nedergaard et al., 2005), while PPARα is highly expressed in BAT, and it works as a pivotal actor in the regulation of BAT thermogenic activity (Hondares et al., 2011). Both PPARγ agonists and PPARα agonists upregulated the expression of *UCP1* and *PGC1*α in brown adipocytes (Barbera et al., 2001; Hondares et al., 2006, 2011), which were mediated by PPARs' binding to the PPAR (Figure 1). Similar effects of PPAR agonists were observed within WAT. PPARγ agonists such as thiazolidinediones induced the browning of WAT *in vitro* and *in vivo*, demonstrated by mitochondrial morphology changes (small lipid droplets surrounded by mitochondrial in white adipocyte), increased mitochondrial mass, β-oxidation of fatty acids and the expression of *UCP1* and *PGC1*α (Wilson-Fritch et al., 2003, 2004; Bogacka et al., 2005). PPARα agonists including Wy14,643 (pirinixic acid), GW6471, and GW7647 induced the browning of WAT as well, accelerating mitochondrial biogenesis and fatty acid oxidation via induction of *PGC1*α and *PRDM16* expression (Ribet et al., 2010; Hondares et al., 2011). Therefore, PPAR agonists may be another potential pharmacological targets to correct metabolism disorder.

### Modulation of thermogenic adipose tissue by lifestyle

Excess energy intake over expenditure results in overweight and obesity. Common strategies for weight loss are lifestyle interventions, such as exercise and energy restriction. Thermogenic adipose tissue has been reported as a target to modify energy expenditure. Accumulating data confirmed that lifestyle interventions can activate thermogenic adipose tissue, further enhance energy expenditure (Slocum et al., 2013).

Firstly, dietary intake induces alteration of morphology and *UCP1* expression in BAT. Accompanied with the increased body weight in response to high-fat diet (HFD) feeding, light microscopy disclosed that the morphology of BAT had greater cytoplasmic lipid accumulation with HFD (Faber et al., 2014), which was abolished by time-restricted feeding (Chaix et al., 2014). Moreover, time-restricted feeding blocked the upregulated expression of proinflammatory cytokines/chemokine (*TNF*α, *IL1*β, and *Ccl8*), indicating the role of diet-induced inflammation in BAT dysfunction (Chaix et al., 2014). In addition to it, high calorie diet feeding displayed increased *UCP1* mRNA levels in BAT(Hansen et al., 2014), suggesting an adaptation against the excess of energy intake. Similarly, short-term calorie restriction led to decrease in UCP1 content and mitochondrial differentiation, accompanied with decreased oxygen consumption (Valle et al., 2007).

Secondly, dietary restriction conjunction with exercise induces changes in morphology, genes expression in mitochondria and inflammatory response. The BAT exhibited decreased cytoplasmic lipid droplets and increased mitochondria-rich eosinophilic, mitochondrial cristae, fenestration of mitochondrial cristae, and elongated (lamellar) mitochondria after exercise and dietary restriction (Slocum et al., 2013; Faber et al., 2014). Slocum et al. demonstrated that some genes about mitochondrial biogenesis/functions in BAT were up-regulated after exercise and dietary restriction, indicating the increased mitochondrial functions and explaining the increased energy expenditure (Slocum et al., 2013). These genes including nuclear respiratory transcriptional factor 1, guanine andadenine-binding protein alpha, mitochondrial transcription factor A, *PGC1*α, and protein in kinase AMPK-activated alpha 1, BAT β-oxidation pathway enzymes (acyl-Coenzyme A dehydrogenase family, member 11 and thioesterase superfamily member 2) and fatty acid transporters [fatty acid-binding protein 3, acetyl-Coenzyme A acyltransferase 1B, carnitine palmitoyltransferase I, and solute carrier family 27 (fatty acid transporter), member 2 fatty acid transport; Table 2].

**Table 2:** **Exercise and diet restriction induce gene expression changes in brown adiopose tissue**.

**Gene symbol**	**Exercise**		**Diet-restriction**	
	**Log 2 fold change**	***P*-value**	**Log 2 fold change**	***P*-value**
UCP1	2.1020976	0.003	1.6475139	0.492
NRF1	1.0239830	0.043	1.3430682	0.009
GABPA	1.0370150	0.018	2.078541	7.42E-06
TFAM	1.3344975	0.007	2.5830269	1.19E-06
PGC1α	0.9481345	0.024	1.8143594	0.492
PRKAA1	0.9179600	0.042	1.8682966	0.0001
ACAD11	0.8625050	0.030	1.0620409	0.009
THEM2	1.4237638	0.010	2.2965239	0.0001
FABP3	0.9796286	0.042	1.5966204	0.001
ACAA1B	1.5523714	0.023	1.9453986	0.005
CPT1B	0.9934236	0.010	1.2340546	0.002
SLC27A2	1.2564735	0.030	1.3538418	0.020

UCP1, uncoupling protein 1; NRF1, nuclear respiratory factor 1; GABPA, guanine and adenine-binding protein alpha; TFAM, mitochondrial transcription factor A; PGC1α, peroxisome proliferator activated receptor-γ coactivator-1α; PRKAA1, protein in kinase, AMPK-activated, alpha 1; ACAD11, acyl-Coenzyme A dehydrogenase family, member 11; THEM2, thioesterase superfamily member 2; FABP3, fatty acid-binding protein 3; ACAA1B, acetyl-Coenzyme A acyltransferase 1B; CPT1B, carnitine palmitoyltransferase I; SLC27A2, solute carrier family 27 (fatty acid transporter), member 2 fatty acid transport.

Thirdly, exercise increases *UCP1* expression and brown adipocyte progenitor cells. It has been shown that mitochondrial *UCP1* was up-regulated in the BAT after exercise (Slocum et al., 2013; Table 2). In addition, exercise, under both normal diet and HFD-feeding condition, increased the population of brown adipocyte progenitor cells and the levels of *UCP1* in BAT, both of which serve as the structural foundation for energy expenditure (Xu et al., 2011b). Taken together, these changes demonstrated that BAT activation was elicited by exercise, thereby ameliorating the metabolic abnormality.

Forthly, exercise induces the browning of WAT. It is well known that exercise induces a series of well-recognized beneficial effects in the muscle (Handschin and Spiegelman, 2008). Some compelling findings also demonstrated that exercise drove browning of WAT in mice and human (Xu et al., 2011b; Bostrom et al., 2012; Moreno-Navarrete et al., 2013). For instance, exercise training increased mitochondrial number and brown adipocyte-specific gene expression in eWAT in mice (Xu et al., 2011b), including *UCP1, PGC1*α, *Dio2* (converting the prohormone thyroxine by outer ring deiodination to bioactive 3,3′,5-triiodothyronine) and *C/EBP*β. Furthermore, exercise increased the secretion of protein and small molecules in the muscle, followed by releasing into the circulation and entering iWAT. With gene expression arrays and a bioinformatics technique, irisin from muscle was found and identified as a polypeptide hormone, with the effect of inducing WAT browning and enhancing thermogenesis, further inhibiting diet-induced obesity, and insulin resistance. The mechanism of increasing secretion of irisin is that muscle PGC-1α generation induced by exercise increased the fibronectin-type III domain-containing 5 expression, and then further upregulated irisin production (Bostrom et al., 2012; Moreno-Navarrete et al., 2013). With liquid chromatography-mass spectrometry (LC-MS) metabolic profiling technique,β-aminoisobutyric acid (BAIBA)was recently highlighted as a novel small molecules in the muscle by PGC-1α mediated mechanism, with the effect of elevation of brown adipocyte-specific genes (*UCP1, Cidea*, and *PRDM 16*) in WAT via PPARα dependent pathways, further improving glucose tolerance and inhibiting weight gain (Roberts et al., 2014). Therefore, irisin and BAIBA could be a potential therapeutic approach to protect against obesity-associated diseases.

Finally, dietary factors are associated with the browning of WAT too. However, the relevant results are controversial. In some studies with mice, expression of *UCP1* in the iWAT and eWAT was lower in the HFD group than normal diet group (Rong et al., 2007; Kim and Park, 2010). Contrary to it, *UCP1* expression in iWAT and retroperitoneal WAT (rWAT) was increased in HFD/high protein/energy diet group compared with normal diet/low protein/energy diet group in rats (Hojna et al., 2012) and bovine (Asano et al., 2013). Whether this difference in results is due to species specificity merit further confirmation.

### Inhibiton of thermogenic activity by environmental pollutants

Recently, major environmental pollutants like air pollution and ozone (O_3_) are connected with metabolic diseases (Sun et al., 2009, 2013). The particulate matters are the major pollutants in the air. According to the aerodynamic diameter, particles are named as PM_10_ (with a diameter less than 10 μm), PM_2.5_ (with a diameter less than 2.5 μm), PM_0.5_ (with a diameter less than 0.5 μm). Some of the characteristics of PM_2.5_, including the small size, chemical composition, and potential ability to filtrate into the circulation, make it as the main factor jeopardizing human health. Exposure to environmental PM_2.5_ induces a series of metabolic disorders such as glucose tolerance, insulin resistance, thermogenic adipose tissue dysfunction, increased body weight gain and ultimately metabolic diseases (Sun et al., 2009, 2013; Xu et al., 2010; Liu et al., 2014c).

#### Environmental pollutants inhibit BAT function

Recent studies demonstrated that exposure to PM_2.5_ reduced O_2_ consumption/CO_2_ production and heat production (Xu et al., 2011a; Liu et al., 2014b), indicating that PM_2.5_ induce energy metabolism disorder. As the crucial organ for energy metabolism, BAT arouse extensive concern regarding air pollution-induced metabolic diseases. Studies have shown that PM_2.5_ exposure significantly decreased the weight of BAT and resulted in a significant reduction in mitochondrial number and average mitochondrial size (Xu et al., 2011a,c, 2012). The adipocyte-specific gene profiles in BAT were significantly reduced in response to PM_2.5_ exposure. These genes include *UCP1, PGC1*α, *Dio2, Cidea*, and *Elovl3* (an elongase enzyme important for elongation of monounsaturated or saturated very long chain fatty acids; Xu et al., 2011a,c, 2012). These results indicate that exposure to PM_2.5_ may induce BAT dysfunction.

#### Environmental pollutants induce BAT inflammation

Exposure to PM_2.5_ was observed to increase expression of interleukin-6 (IL-6) and tumor necrosis factor (TNF)-α in BAT, suggesting PM_2.5_-induced inflammation in BAT (Liu et al., 2014a). TNFα is a strong pro-inflammatory cytokine that plays an important role in the immune response (Zelova and Hosek, 2013) and it has been shown to negatively regulate BAT development by inhibiting proliferation (Porras et al., 1997), differentiation (Valladares et al., 2001), apoptosis (Porras et al., 1997), and inducing morphological changes (Porras et al., 1997) in brown adiocytes. TNFα also appeared to be partially or completely responsible for the apoptosis of brown adipocytes *in vitro* (Valladares et al., 2000) which was enhanced by p38 MAPK, whereas attenuated by extra-cellular-regulated kinases (ERKs; Valladares et al., 2000). Thus, PM_2.5_ exposure may impact BAT development and apoptosis through TNFα mediated inflammation. The direct evidence still remains further exploration.

The increased TNFα and IL-6 expression in the hypothalamus of animal models exposed to PM_2.5_ indicates that PM_2.5_ inhalation induced central inflammation (Ying et al., 2014; Liu et al., 2014b). Based on the results that inhalation of PM_2.5_ down-regulated *UCP1* expression in BAT (Xu et al., 2011a) and that BAT functional activation is an important target for hypothalamic inflammation (Holt et al., 1989; Arruda et al., 2010, 2011), it leads us to investigate the role of hypothalamus inflammation in BAT dysfunction. Studies have shown that high concentration of hypothalamic TNFα upregulated the expression of *UCP1* in BAT (Arruda et al., 2010), whereas low concentration of hypothalamic TNFα downregulated the expression of *UCP1* (Arruda et al., 2011). These results suggest the dual effect of hypothalamic TNFα in BAT function, increasing the activation of BAT at high concentration (Arruda et al., 2010) and decreasing the BAT thermogenic activity at low concentration (Arruda et al., 2011). Thus, to elucidate the different effect of central TNFα at different levels on BAT function is important to explain the role of PM_2.5_-induced hypothalamus inflammation in metabolic disorder.

Researchers have studied the different effect of central TNFα at different levels on BAT in details. In one hand, a high-concentration of intracerebroventricular TNFα (icv intervention) activated the sympathetic tonus through β_3_AR, increasing UCP1 and *PGC1*α expression and inducing mitochondrial biogenesis in the BAT. The enhanced expression of thermogenic genes and mitochondrial function lead to increased body temperature and whole-body O_2_ consumption/CO_2_ production, resulting in the loss of body mass, and reduced size of lipid droplets (Arruda et al., 2010). In the other hand, a low dose of icv TNFα, mimicing some of the features of low-grade/obesity-like hypothalamic inflammation, reduced UCP1 and PGC1α expression and inhibiting mitochondrial biogenesis in the BAT and resulted in reduced O_2_ consumption/CO_2_ production (Arruda et al., 2011). In line with the observations of low dose TNFα administration, deletion of TNF receptor 1 (TNFR1), a main TNFα receptor, were protective against diet induced obesity by increasing BAT activation and enhancing thermogenesis (Romanatto et al., 2009). It has been reported that there are two kinds of TNFRs, TNFR1, and TNFR2 with an opposite function (Chen and Palmer, 2013). TNFα at high concentration may activate both TNFR1 and TNFR2, with effects of TNFR1 concealed by that of TNFR2, rending increased BAT thermogenic activity and obesity. However, TNFα at low concentration may only activate TNFR1 and only the TNFR1-mediated effect (decreased BAT function) was demonstrated. It is interesting to observe that TNFα antibody administration by icv even exaggerated PM_2.5_-induced energy metabolism dysfunction, which may be associated with the concentration of central TNFα. However, central inhibition of IKK2 with IMD-0354 did show protective effect on the PM_2.5_-induced peripheral inflammation and metabolic disorder, including O_2_ consumption, CO_2_ production, heat production, glucose tolerance, and insulin sensitivity (Liu et al., 2014a,b). Because it is hard to identify the proper concentration of TNFα in the hypothalamus, these results could not hinder us from the conclusion that exposure to PM_2.5_ may induce low-grade inflammation in the hypothalamus and BAT dysfunction.

It has been reported that EAT highly expresses BAT specific genes (Sun et al., 2013) and demonstrated some characteristics of the thermogenic adipose tissues. In addition to the decreased gene expression of *UCP1, PGC1*α, and *Cidea* (Sun et al., 2013), exposure to PM_2.5_ and/or O_3_ increased adipose tissue macrophages and resulted in pro-inflammatory macrophages shift by an increase in *TNF*α, *IL-6* and a decrease in *IL-10, MgI1* gene expression in EAT (Sun et al., 2013). However, the function and specific characteristics of EAT are far from clear and the influence of air pollution on it remains further investigation.

#### Environmental pollutants induce BAT insulin resistance

BAT is a target tissue for insulin action, especially during late fetal development when insulin promotes adipogenic and regulates glucose uptake (Valverde et al., 2005). Studies have shown that long-term exposure to PM_2.5_ impairs insulin signaling in BAT demonstrated by decreased level of phosphorylation of AKT at ser473 (Xu et al., 2011a). The occurrence of insulin resistance in BAT may due to inflammation to some extent. In our previous studies, the expression in inflammatory genes in BAT (such as *TNF*α, *IL-6*, and *F4/80* etc.) increased in response to PM_2.5_ inhalation(Liu et al., 2014a), whereas, TNFα implicates a link between adiposity and the development of insulin resistance and is an important contributor to the pathogenesis of type 2 diabetes (Hotamisligil et al., 1993). For instance, TNFα in brown adipocytes caused impairment in the IRS-2-associated PI3-kinase signaling in response to insulin (Valverde et al., 1998) and inhibited insulin-induced glucose uptake, such as *GLUT4* mRNA expression and GLUT4 translocation to the plasma membrane in the brown adipocytes (Valverde et al., 1998; Teruel et al., 2001).

TNFα-induced insulin resistance in brown adipocytes could be in part mediated by ceramide (Teruel et al., 2001). Ceramide, which is a suppressor of cell growth and an inducer of apoptosis, has been invoked as a mediator of some effects of TNFα. Ceramide is a central molecule in sphingolipid structure and TNFα induced ceramide generation by hydrolysis of sphingomyelin (Hannun and Obeid, 1995). C2-ceramide, a short-chain ceramide analog, mimicked TNF-α to induce insulin resistance, decrease GLUT4 gene expression (Fernandez-Veledo et al., 2006), and completely preclude insulin-stimulated glucose uptake and insulin-induced GLUT4 translocation to plasma membrane (Teruel et al., 2001; Fernandez-Veledo et al., 2006). In addition, C2-ceramide inhibited insulin-stimulated AKT pathway by activating PP2A phosphatase, but not PI3-kinase or PKC-ζ activities (Teruel et al., 2001).

Another mechanism that TNF-α induced insulin resistance in brown adipocytes could be via stress kinases. One study indicated that the activation of p42/p44 and p38 MAPK by TNFα impaired insulin stimulation of IRS-2 associated PI3K, leading to insulin resistance in brown adipocytes (Hernandez et al., 2004). TNFα in the presence of PD98059 or PD169316, inhibitors of p42/p44 and p38 MAPK, respectively, restored insulin signaling and insulin-induced glucose uptake (Hernandez et al., 2004). Previous studies showed that the levels of p38 MAPK and ERK was increased and IRS-1 mediated signaling was suppressed in response to PM_2.5_ inhalation in liver (Zheng et al., 2013; Liu et al., 2014a,c). However, the direct evidence of PM_2.5_-activated MAPK activity in BAT requires further research.

#### Environmental pollutants induce oxidative stress and mitochondrial dysfunction in BAT

Mitochondrial function has been indicated to play a major role in type 2 diabetes mellitus (Jelenik and Roden, 2013). Previous literature indicated that exposure to PM_2.5_ resulted in oxidative stress and mitochondrial dysfunction in BAT Xu et al., 2011a,c; Liu et al., 2014a. Exposure to PM_2.5_ for 2 months increased ${\text{O}}_{2}^{-}$ in BAT in ApoE^−∕−^ mice, indicating PM_2.5_ exposure could trigger reactive oxygen species production (Xu et al., 2011c). Consistent with it, long term PM_2.5_ exposure in C57BL/6 for 10 months demonstrated significantly increased superoxide and higher expression of 3-nitrotyrosine in BAT, increased expression of Phase II antioxidant genes, such as NF-E2-related factor 2, NAD(P)H quinone oxidoreductase 1, and glutamate-cysteine ligase modifier subunit (Xu et al., 2011a). In addition, mitochondrial number and size were significantly reduced in the BAT in response to PM_2.5_ exposure and/or O_3_ exposure Xu et al., 2011a,c; Sun et al., 2013, which may be caused by ROS production.

## Conclusions

Usually, obesity is ascribed to excess energy intake and storage, with WAT accumulation as the major visible alteration. It is well known that obesity is obviously related to insulin level, metabolic syndromes, and cardiovascular diseases, to which WAT accumulation and BAT dysfunction contributed tremendously. The issue of how to control the explosive obesity crisis has been investigated for several decades. Much more attention has been paid to accelerating energy expenditure as a strategy to control obesity and related metabolism diseases. Accumulating evidence indicates that thermogenic adipose tissues, including BAT and browned WAT (beige/brite fat), are also present in adult humans and play a critical role in energy expenditure and whole-body metabolism. The activation of both BAT and beige adipocytes by cold stimulation, medication or lifestyle can improve insulin sensitivity and potentially protect against obesity and other metabolic diseases. β_3_AR and PPRE seems to be one of the central link in thermogenic adipose tissue activity. Many of the thermogenic inducers, such as irisin and BAIBA, may produce tremendous interest to activate thermogenic adipose tissue function. Thus, it may work as a therapeutic tool to cure obesity and metabolic diseases in the future. However, how to effectively and safely stimulate and activate thermogenic adipose tissue in humans is poorly understood. Furthermore, exposure to environment pollutants induces thermogenic adipose tissue dysfunction, further aggravating metabolic diseases. Since air pollution has become a significant public health challenge in many countries, better knowledge on these adipose abnormality and mechanisms involved in PM_2.5_-mediated thermogenic adipose tissue dysfunction would help to identify the preventative or targeted therapies.

## Author contributions

GZ contributed to the design of the work, drafting the work, QS contributed to the concept of the work, revising the work, CL contributed to the concept and the design of the work, revising the work. All authors approved the version to be published, and agreed to be accountable for all aspects of the work in ensuring that questions related to the accuracy or integrity of any part of the work are appropriately investigated and resolved.

## Funding

This work was supported by NIH ES018900 (to QS), National Natural Science Foundation of China 81402646, Zhejiang Provincial Natural Science Foundation of China LQ13H070002, Hangzhou Science & Technology Plan Project of China 20140633B36 and the Scientific Research Foundation for the Returned Overseas Chinese Scholars, Ministry of Education of China (to CL).

### Conflict of interest statement

The authors declare that the research was conducted in the absence of any commercial or financial relationships that could be construed as a potential conflict of interest.

## Abbreviations

WAT: white adipose tissue
BAT: brown adipose tissue
UCP1: uncoupling protein 1
C/EBP: CCAAT-enhancer binding protein
PPARγ: peroxisome proliferator-activated receptor γ
PPARα: peroxisome proliferator-activated receptor α
FDG: fluoro- D-glucose
iBAT: interscapular brown adipose tissue
PRDM16: PRD1-BF1-RIZ1 homologous domain containing
β_3_AR: β_3_-adrenergic rectptor
iWAT: inguinal subcutaneous WAT
eWAT: epidydimal WAT
Sca-1: stem cell antigen 1
PDGFRα: platelet-derived growth factor receptor alpha
EAT: epicardial adipose tissue
PGC1α: proliferator-activated receptor-γ coactivator 1α
cAMP: cyclic AMP
p38 MAPK: p38 mitogen-activated protein kinase
CREB: cAMP response element binding protein
PPARs: peroxisome proliferator-activated receptors
rWAT: retroperitoneal WAT
PM: particulate matters
TNF: tumor necrosis factor
TNFR: TNF receptor.
